# A Mathematical Model of In Vitro Cellular Uptake of Zoledronic Acid and Isopentenyl Pyrophosphate Accumulation

**DOI:** 10.3390/pharmaceutics14061262

**Published:** 2022-06-14

**Authors:** Elena Lo Presti, Laura D’Orsi, Andrea De Gaetano

**Affiliations:** 1 CNR-IRIB (Institute for Biomedical Research and Innovation), National Research Council, Via Ugo La Malfa 153, 90146 Palermo, Italy; 2 CNR-IASI BioMatLab (Institute of Analysis, Systems and Computer Science), National Research Council, Via dei Taurini 19, 00185 Rome, Italy; laura.dorsi@biomatematica.it

**Keywords:** zoledronic acid, gamma-delta T cells, immunotherapy, isopentenyl pyrophosphate, ATRAID, pharmacodynamics, mathematical modeling, parameters estimation

## Abstract

The mevalonate pathway is an attractive target for many areas of research, such as autoimmune disorders, atherosclerosis, Alzheimer’s disease and cancer. Indeed, manipulating this pathway results in the alteration of malignant cell growth with promising therapeutic potential. There are several pharmacological options to block the mevalonate pathway in cancer cells, one of which is zoledronic acid (ZA) (an N-bisphosphonate (N-BP)), which inhibits the farnesyl pyrophosphate (FPP) synthase enzyme, inducing cell cycle arrest, apoptosis, inhibition of protein prenylation, and cholesterol reduction, as well as leading to the accumulation of isopentenyl pyrophosphate (IPP). We extrapolated the data based on two independently published papers that provide numerical data on the uptake of zoledronic acid (ZA) and the accumulation of IPP (Ag) and its isomer over time by using in vitro human cell line models. Two different mathematical models for IPP kinetics are proposed. The first model (Model 1) is a simpler ordinary differential equation (ODE) compartmental system composed of 3 equations with 10 parameters; the second model (Model 2) is a differential algebraic equation (DAE) system with 4 differential equations, 1 algebraic equation and 13 parameters incorporating the formation of the ZA+enzyme+Ag complex. Each of the two models aims to describe two different experimental situations (continuous and pulse experiments) with the same ZA kinetics. Both models fit the collected data very well. With Model 1, we obtained a prevision accumulation of IPP after 24 h of 169.6 pmol/mgprot/h with an IPP decreasing rate per (pmol/mgprot) of ZA ($k_{XGZ}$) equal to 13.24/h. With Model 2, we have comprehensive kinetics of IPP upon ZA treatment. We calculate that the IPP concentration was equal to 141.6 pmol/mgprot/h with a decreasing rate/percentage of 0.051 ($k_{XGU}$). The present study is the first to quantify the influence of ZA on the pharmacodynamics of IPP. While still incorporating a small number of parameters, Model 2 better represents the complexity of the biological behaviour for calculating the IPP produced in different situations, such as studies on γδ T cell-based immunotherapy. In the future, additional clinical studies are warranted to further evaluate and fine-tune dosing approaches.

## 1. Introduction

The mevalonate pathway represents the most important metabolic pathway of the cell system for synthesizing bioactive molecules involved in growth control or the synthesis of cholesterol [1]. Furthermore, the existence of several pathological conditions [2,3,4] due to defects in the pathway is proof of its importance. Although the main regulatory point in the mevalonate pathway is 3-hydroxy-3-methylglutaryl coenzyme A reductase (HMGR), one of the most highly regulated enzymes in the body [1,5], the FPPS enzyme plays a crucial role in the pathway. In fact, after the mevalonate is converted to isopentenyl pyrophosphate (IPP) by the action of a cascade of three enzymes, FPPS combines the GPP (geranyl pyrophosphate) with another molecule of IPP to produce farnesyl pyrophosphate (FPP). This further reacts with IPP to form geranylgeranyl pyrophosphate (GGPP). While FPP is a major branch-point precursor for sterols with an end-point in cholesterol production, FPP, together with GGPP, is the focal point in the non-sterol branch, which is the basis of post-translational modifications of small GTP-binding proteins (Ras, RhoA, Rac, etc.) [6].

The mevalonate pathway is an attractive target in many therapeutic research areas concerning autoimmune disorders, atherosclerosis, and Alzheimer’s disease [7]. The manipulation of this pathway results in alteration of malignant cell growth with promising potential for application in human cancers [8]. Indeed, several pharmacological options block the mevalonate pathway in cancer cells, such as statins and amino-bisphosphonates (N-BPs). The former inhibits the early phase of the pathway, and the latter inhibits the FPP synthase enzyme, inducing cell cycle arrest, apoptosis, inhibition of protein prenylation, and cholesterol reduction [9,10,11,12,13,14]. Mevalonate pathway inhibitors, in combinations among themselves and with cytotoxic drugs, represent a promising approach to enhancing the efficacy of anticancer therapies [8].

Three generations of BPs with increasing anti-resorptive potency have been successively developed. The most active compound of this class is zoledronic acid (ZA), which is up to 10,000 fold more potent than first-generation compounds and has been used successfully in clinical applications [15]. The research about the pharmacokinetics of BPs administered as drugs have demonstrated that BPs, in blood, are bound to albumin and are quickly cleared from plasma, with about 50 percent deposited in bone and the remaining part excreted in the urine. Tumor cells uptake BPs very rapidly by fluid-phase endocytic internalization [16], and in parallel, they bind to Ca${}^{2+}$-containing bone mineral surfaces at sites of active bone remodeling [17,18]. After bone resorption, osteoclasts take BPs in endocytic vacuoles [19], and their acidification, together with the assembly of the complex ATRAID-SLC37A3, are required for BPs to enter the cytosol, thereby allowing either the diffusion or transport of BPs across the vesicular membrane [16,20].

When zoledronic acid (ZA) is in the cytosol, it binds FPPS, thus inhibiting the transformation of the early metabolites of the mevalonate pathway (IPP) in GPP, resulting in the accumulation of IPP [21] and its isomer DMAPP [22]. ZA shows an immunomodulatory effect on the immune system and is being widely discussed in this pandemic period (COVID-19). Indeed, as an immunostimulant, it boosts anti-viral and anti-tumoral γδ T cell expansion thanks to the accumulation of IPP [23]. At the same time, it could act on dendritic cells (DC) to stimulate the immune response by modifying the expression of functional markers such as CD83, HLADR, and CD80 [24]. The exact mechanisms through which Vγ9Vδ2 T cells become activated by IPP are a recent topic of investigations [25,26]. Upon IPP activation, Vγ9Vδ2 T cells produce and release pro-inflammatory cytokines (such as tumor necrosis factor-alpha (TNF-α), chemokines, interferon-γ (IFN-γ)) and, with the addition of IL2, Vγ9Vδ2 proliferates and also acquires cytotoxic functions. As mentioned before, considering the immuno-modulatory function of ZA, extensive evidence from preclinical studies [27] showed that ZA exerts its anticancer actions in different ways: (1) by a direct effect on tumor cells, inhibiting proliferation and inducing apoptosis in vitro (with sensitivity to this effect largely depending on the ability of tumor cells to internalise sufficient amounts of N-BP to inhibit FPPS); (2) indirectly by affecting bone resorption, thereby reducing tumor cell migration to the bone [28]; (3) inducing IPP production by tumor cells and tumor-associated macrophages (TAM) that activate cytotoxic Vγ9Vδ2 T cells against the tumor; (4) as very recently demonstrated [29], by causing the shift of TAM in the M1 phenotype; (5) by inhibiting the adhesion of tumor cells to the extracellular matrix proteins and, thus, by impairing the process of tumor cell invasion and metastasis [30].

Several theoretical models have been developed in recent years to further refine treatment regimens for cancer patients [31]. Typically, cellular response to a drug is often evaluated by a variety of in vitro assays and is generally interpreted using dose-response curves. In these assays, the drug is typically applied to a cell population over a wide range of concentrations and evaluated over time (usually 72 h) to test the effects on cell death (often indirectly). Based on in vitro experiments with ZA, several clinical trials for patients with different types of tumors have been performed [32,33]. Small changes in experimental timing or pharmaceutical concentrations appear to significantly impact the outcomes [34]. Several methods have been used to quantify ZA and IPP based on radioactive tracing, gas chromatography-mass spectrometry (GC-MS), or by liquid chromatography-tandem MS (LC-MS/ MS) [35]. An in vitro approach could be used to support the formulation of mathematical models, with the main aim of providing quantitative tools describing the pharmacokinetics and pharmacodynamics (PKPD) of the compound under investigation [31]. Verhulst et al. [36] proposed an in vitro model of primary human tubular kidney cells to mimic the most important physiological characteristics of molecular uptake/transport by the tubular epithelium *in vivo*. At present, the pharmacokinetics of ZA have been assessed on clinical data of patients with bone metastases from a variety of primary cancers. The relationship between the dose and drug safety is also studied [17,37]. However, while in the studies mentioned above, the temporal relationship between ZA-treatment and its effects has received great attention from different viewpoints, to date, a model describing the PKPD of ZA and IPP is missing.

Such a model would be very useful due to recent evidence supporting the generation of optimised protocol to expand efficient Vγ9Vδ2 T cells [38], with enhanced activation and differentiation of human Vγ9Vδ2 T cells upon the restimulation of short-term-expanded γδ T cell lines with L ascorbic acid 2 phosphate (pVC). Attempts to find novel approaches to expand Vγ9Vδ2 T cells, approaches that could be transferred to the clinical care of cancer patients, would benefit from the quantitative indications that such a model could provide.

Therefore, the general objective of the present study is to describe a (possibly simple) mathematical model that satisfactorily describes the dynamics of the process of ZA-induced IPP accumulation in cell cultures in vitro, predicting the IPP accumulation in different hypothetical in vitro experiments, and likely to provide a background understanding of the process to eventually represent quantitatively ex vivo ZA administration procedures, such as those used to expand Vγ9Vδ2 lymphocytes for the immunotherapy of cancer patients. We will use previously published experimental data [39,40] from publications which might be considered the first pioneering studies of the kinetics of ZA uptake by tumor cells.

## 2. Materials and Methods

### 2.1. In Vitro Model Assay

We extrapolated the observations from two independently published papers [39,40], which provided numerical data on the uptake of ZA and the accumulation of IPP (Ag) and its isomer over time by using in vitro human cell line models. The papers’ authors belong to the same institutions, the University of Eastern Finland, INSERM and the University of Lyon, even if the common authors are only Hannu Mönkkönen and Jukka Mönkkönen. The authors chose two different breast cancer cell lines: Raikkonen et colleagues chose the MCF7 cell line, an epithelial breast adenocarcinoma cell line derived from a metastatic pleural effusion. At the same time, Benzaid et colleagues chose T47D. Both grow in a monolayer as epithelial-like cells. MCF7 and T47D uptake ZA in similar amounts, but their uptake is three-fold greater than that of the B02 cell line (also a breast cancer cell line). We considered two different experiments: “pulse” administration and continuous “infusion” administration. During the pulse experiment, the cells were exposed to ZA for 1 h at an initial concentration in the supernatant of 25 μM/mL, after which the supernatant was removed and replaced by a fresh medium. The samples were collected at different time points in Raikkonen’s and Benzaid’s experiments (0 h, 1 h, 3 h, 6 h, 12 h, 18 h, 24 h or 48 h and 0 h, 4 h, 8 h, 12 h, 24 h, 42 h, respectively). For the continuous experiment, cells were treated initially at a concentration in the supernatant of 25 μM/mL, and observed for 1 h, 3 h, 6 h, 12 h, 18 h, 24 h or 48 h; the continuous experiment was available only from Raikkonen’s paper.

The concentration values of ZA and IPP in the cells were assessed through the cell extract preparations described in [41], in which the molar amount of drug per mg protein was determined. The concentration analysis of IPP was performed by high-pressure liquid chromatography (HPLC) negative ion electrospray ionization mass spectrometry (HPLC-ESI-MS) as indicated in [42], while ZA concentrations were evaluated through radioactivity measurements as reported in [39]. The HPLC-ESI-MS analysis appears to be the same for both groups. In fact, the authors declared using the protocol of Monkkonnen’s research group, as well as the level of drug uptake quantified with the equal protocol and tool. Cells were cultured at 37 °C in RPMI-1640 medium supplemented with 10 percent fetal bovine serum and 100.0 (IU · mL${}^{-1}$) penicillin-streptomycin in a 5 percent CO${}_{2}$ atmosphere. Breast cancer cell lines were harvested using 0.250 percent trypsin, and, to evaluate the IPP concentration in both Raikkonen and Benzaid’s experiments, cells were seeded in 6-well plates at a density of $10^{6}$ cells per well and left to adhere overnight. For ZA detection, cells were seeded overnight to 10-cm Petri dishes at $4\cdot 10^{6}$ cells/dish and then treated.

### 2.2. Mathematical Model

In the present section, two different IPP-ZA pharmacokinetic models (PKM), Model 1 and Model 2, are described. The first model (Model 1) is an ordinary differential equation (ODE) system with 3 equations and 11 parameters; the second model (Model 2) is instead a differential algebraic equation (DAE) system with 4 differential equations, 1 algebraic equation and 13 parameters. Both models aim to describe the two different experimental situations (continuous and pulse experiments) with the same ZA kinetics (Equations (1) and (2)) but different formulations for IPP dynamics.

For greater clarity, the biological variables considered (Table 1) will be indicated in the mathematical formulation as follows: *Y* (μM) is the zoledronate concentration in the medium; *Z* (pmol/mg prot) is the zoledronic acid (ZA) intracellular concentration; *G* (pmol/(mg prot)) is the antigen (IPP) concentration; *B* ((%)) is the percentage of bound enzyme and U((%)) is the percentage of unbound enzyme.

**Figure 1:**
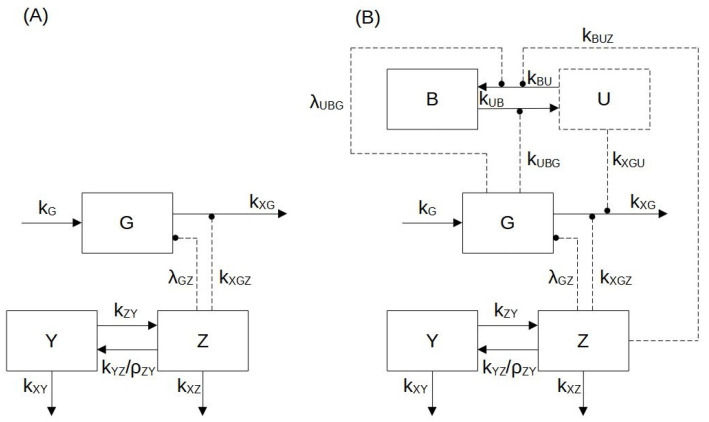
Block diagram of Model 1 (panel **A**) and Model 2 (panel **B**). According to the mathematical formulation used for two models, the biological variables considered are: the zoledronate concentration in the medium (Y(μM)); the zoledronic acid (ZA) intracellular concentration (Z (pmol/mg prot)); the antigen (IPP) concentration (G (pmol/(mg prot))); the percentage of bound enzyme (B (%)) and the percentage of unbound enzyme (U (%)). Blocks with a continuous contour represent differential variables (Z, Y, G, B), and the block with a dashed contour represents the algebraic variable (U). Arrows in continuous lines indicate mass transfer, while dashed arrows indicate enhancement (arrow endpoints) or suppression (circle endpoints).

Each model variable is represented with a compartment (Figure 1), with transfer rates from one compartment to another being indicated with $k_{ij}$, where *j* represents the compartment of origin and *i* represents the arrival compartment.

Zoledronate kinetics are described with the same equations (Equations (1) and (2)) in both models (Model 1 and Model 2).

The supernatant (medium) ZA concentration *Y* [μM] variation over time is:

(1) $$\frac{dY\left(t\right)}{dt}=\;\;-\left(k_{ZY}+k_{XY}\right)\;\;Y\left(t\right)\;\;\;-\;\;\;\chi_{pulse}\;\;\delta \left(t-t^{*}\right)\left[Y\left(t\right)-Y^{*+}\right]\;\;\;+\;\;\;\frac{k_{YZ}}{\rho_{ZY}}\;\;\;Z\left(t\right),\;\;\;\;\;\;Y\left(t_{0}\right)\;\;=\;\;25\;\;\left[\mu M\right]$$

where $k_{ZY}$ (/h) is the ZA transfer rate from medium to cells; $k_{XY}$ (/h) is the medium ZA loss (degradation) rate; $k_{YZ}$ (/h) is the transfer rate from cell ZA to medium ZA; and $\rho_{ZY}$ (pmol/mgprot/μM) is the parameter converting μM to pmol/mgprot.

$\chi_{pulse}$ is an indicator variable that distinguishes the type of experiment:

$$\chi_{pulse}=\left\{\begin{array}{cc} \;\;0 & \;\;continuous\;\;\;experiment\\ \;\;1 & \;\;pulse\;\;\;experiment. \end{array}\right.$$

$Y^{*+}$ (μM) is the ZA concentration in the medium after the removal occurring at time instant $t^{*}$ (after one hour).

*Z* (pmol/mgprot) is the ZA concentration in the cells, and its variation over time is:

(2) $$\frac{dZ\left(t\right)}{dt}=\;\;\rho_{ZY}\;\;k_{ZY}\;\;Y\left(t\right)\;\;\;-\;\;\;\left(k_{XZ}+k_{YZ}\right)\;\;Z\left(t\right),\;\;\;\;\;\;Z\left(t_{0}\right)\;\;=\;\;0$$

where $k_{XZ}$ (/h) is the within-cell ZA loss or degradation rate.

The pharmacodynamics of ZA, i.e., the effect of ZA on within-cell antigen concentrations (IPP), is formalized differently in the two models.

The first model does not consider the formation of a complex made up of ZA + FPPS + IPP (Equation (3)), which is conversely taken into account in the second model in Equations (4)–(6).

The antigen concentration (*G* (pmol/mgprot)) variation over time for Model 1 is:

(3) $$\frac{dG\left(t\right)}{dt}=\;\;k_{G}\;\;\;-\;\;\;\left(k_{XG}+k_{XGZ}\;\;e^{-\;\;\lambda_{GZ}\;\;Z\left(t\right)}\right)\;\;G\left(t\right),\;\;\;\;\;\;G\left(t_{0}\right)\;\;=\;\;G_{0}$$

where $k_{G}=\left[k_{XG}+k_{XGZ}\right]\;G_{0}\;\;=k_{XG}^{tot}\;G_{0}$ (pmol/mgprot/h) is the antigen production rate (assumed constant over the time of the experiments); $k_{XG}$ (/h) is the minimal, irreducible elimination rate of antigen even at infinite ZA concentrations (Z(t)→∞); $k_{XGZ}$ (/h) is the (maximum) antigen elimination rate that can be suppressed (down to zero) in the presence of ZA; and $\lambda_{GZ}$ (/pmol/mgprot) is the rate of exponential decay of antigen elimination rate with increasing ZA concentrations.

In Model 2, the enzyme dynamics are described by Equations (4) and (5), which represent the enzyme’s bound and unbound percentages, respectively:

(4) $$\frac{dB\left(t\right)}{dt}=\;\;k_{BUZ}\;\;Z\left(t\right)\;\;U\left(t\right)\;\;\;-\;\;\;k_{UBG}\;\;e^{-\;\;\lambda_{UBG}\;\;G\left(t\right)}\;\;B\left(t\right),\;\;\;\;\;\;B\left(t_{0}\right)\;\;=\;\;0$$

(5) $$U\left(t\right)=\;\;U\left(t_{0}\right)\;\;-\;\;B\left(t\right),\;\;\;\;\;\;U\left(t_{0}\right)=100$$

where $k_{BUZ}$ (/h/(pmol/mgprot)) is the second order bound enzyme formation rate, depending on both zoledronate and unbound enzyme availability; $k_{UBG}$ (/h) is the maximum bound enzyme dissociation rate; and $\lambda_{UBG}$ (/(pmol/mgprot)) is the rate of exponential decay of the bound enzyme dissociation rate with increasing IPP antigen concentrations.

The IPP concentration variation over time for Model 2 is thus represented as follows:

(6) $$\frac{dG\left(t\right)}{dt}=\;\;k_{G}\;\;\;-\;\;\;\left(k_{XGU}U\left(t\right)+k_{XG}\right)\;\;G\left(t\right)\;\;,\;\;\;\;\;\;G\left(t_{0}\right)\;\;=\;\;G_{0}$$

where $k_{G}=\left(k_{XGU}*U\left(t_{0}\right)+k_{XG}\right)\;G_{0}=k_{XG}^{tot}\;G_{0}$ (pmol/mgprot/h) is once again the antigen production rate (assumed constant over the time of the experiments); $k_{XG}$ (/h) is again the minimal, irreducible elimination rate of antigen even at zero percent unbound enzyme (U(t)→0); and $k_{XGU}$ (/h/%) is the rate of decrease in antigen per percent of unbound enzyme.

Notice that the parameters $k_{G}$, $k_{XG}$ and $k_{XG}^{tot}$ have the same meaning in the two models, but are estimated or computed in a model-specific way in each case.

### 2.3. Parameter Estimation

The model was implemented in C++ (Microsoft Visual Studio 2017 Community Edition), MATLAB (Mathworks MATLAB 2009b) and PHP, using a fixed-step, fourth-order Runge–Kutta numerical integration scheme [43]. The model-free parameters were estimated by ordinary least squares (OLS), using the MATLAB fminsearch” routine for optimization.

The loss function considered is:

$$J\left(\theta \right)=\sum_{t=1}^{N}({\left(Z_{exp}\left(t\right)-Z_{sim}\left(t\right)\right)}^{2}+({\left(G_{exp}\left(t\right)-G_{sim}\left(t\right)\right)}^{2},$$

where θ represents the estimated parameter vector, *N* is the number of experimental observations considered for the normalized sum of squares evaluation, $X_{exp}\left(t\right)$ is the measurement of the observed variable at time *t* and $X_{sim}\left(t\right)$ is the corresponding value obtained by model simulation (*X* indicates a generic observed variable).

The used data points, which represent discrete measurements of zoledronic acid and antigen IPP concentration, were collected by Raikkonen’s [39] and Benzaid’s [40] as described in Section 2.1.

The θ vector is different in two model formulations. In particular,

$$\theta_{1}=\left[k_{ZY},k_{XY},k_{YZ},\rho_{ZY},k_{XZ},k_{XG},k_{XGZ},G_{0},\lambda_{GZ}\right],$$

is the vector of the parameters to be estimated in Model 1 and

$$\theta_{2}=\left[k_{ZY},k_{XY},k_{YZ},\rho_{ZY},k_{XZ},k_{XG},k_{XGU},G_{0},\lambda_{UBG},k_{BUZ},k_{UBG}\right],$$

is the corresponding parameter vector for Model 2. All the values of the estimated parameters in $\theta_{1}$ and $\theta_{2}$ are shown in Table 2 and Table 3.

The complete set of experimental data (48 h for the continuous and 49 h for the pulse experiments) were used for the parameter estimation procedure.

## 3. Results

Our mathematical model consists of simple equations that describe ZA and IPP accumulating into tumor cell lines, by taking into account the experimental data reported in Raikkonen [39] and Benzaid [40]. To evaluate the pharmacokinetics of ZA and the related accumulation of IPP, we referred to two different types of experiments on MCF7 and T47D cells, which we indicate as “pulse” and “continuous” ZA treatments, respectively. More precisely, in the pulse experiment, the tumor cells were treated with ZA for just 1 h, after which the drug-containing medium was removed from the well; in the continuous experiment, the medium was not replaced, and the cells were exposed continuously to the administered ZA. The continuous experiment design is, in fact, closer to in vitro assay applications and to clinical practice. To better understand the pharmacodynamics of drugs and IPP, we propose two different, simple mathematical models of ZA kinetics and IPP dynamics. Model 1 can be considered a basic model, which describes the mechanisms of transfer of ZA from the medium to the cells and its effect on the accumulation of IPP with just three compartmental equations. Model 2 is slightly more comprehensive and considers the molecular mechanisms and the molecular interactions at the basis of the immunomodulation of a particular subset of unconventional T cells.

We note that the observed IPP concentrations from the two experiments were of different magnitude, as could be expected given the limited exposure to ZA in the pulse experiment and the consequently limited effect of the drug on IPP accumulation.

### 3.1. Model 1, Continuous Experiment

In the case of the continuous experiment, only the data by Raikkonen et al. [39] are available. The cells were treated with 25 μM ZA initially and left untouched for 48 h. The intracellular concentration of ZA was determined by comparing the radioactivity of the medium, washes and cell extracts relative to the amount of protein in the cell extract. In this case, the amount of IPP increased gradually with the increasing concentration of ZA in the cells over time. Therefore, the highest intracellular concentrations were achieved at 48 h of ZA exposure and corresponded to 1624 pmol/mgprot of IPP in cells.

Notice that the indicator variable $\chi_{pulse}=0$ (Equation (1)) in this experimental situation, as mentioned in the MM section above.

**Figure 2:**
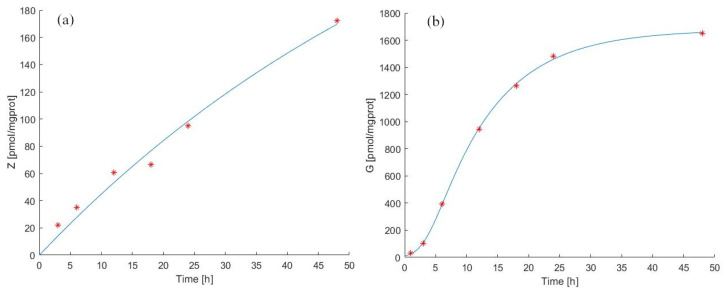
Model 1 simulation trend in the continuous experiment. Panel (**a**) shows the simulated zoledronate (ZA) trend over time; panel (**b**) shows the simulated antigen (IPP) trend over time. The continuous blue line represents the model forecast, while red asterisks represent the experimental data obtained by [39].

Figure 2 shows the time course of the ZA (panel (a)) and IPP (panel (b)) starting from t=0 to t=48 h. The continuous light blue curve represents the predicted time courses as derived by Model 1 Equations (2) and (3), whereas red asterisks indicate observations [39]. The model fits the observed data very well. In response to the administration of ZA, there is an increase in the level of IPP, described by a sigmoid-like curve with a slow initial increase and near steady-state at the end. The parameter values estimated from the continuous experiment are reported in Table 2 and Table 4.

In Figure 2(panel (a) and panel (b)), it is possible to note that between 24 h and 48 h there is a variation of intracellular ZA concentration of about 80 pmol/mgprot. There is a more modest increase between 12 h and 24 h (about 30 pmol/mgprot), contrary to what we observe for IPP levels, which increase more between 12 h and 24 h than between 24 h and 48 h.

Based on the evidence of in vivo experimental data [36], it seems reasonable to assume that a value of transfer rate from medium to tumor cells of ZA ($k_{ZY}=0.007$ /h) eight-fold larger than the transfer from the cell to the medium ($k_{YZ}=0.001$ /h) is due to a preferential transport of the molecule through fluid-phase endocytosis into the cytosol [20].

After 24 h, the ZA concentration increased by 33 times compared to its first hour value ($Z\left(t_{24}\right)=33Z\left(t_{1}\right)$, with *t* expressed in hours), and at 48 h, the ZA concentration was twice the value at 24 h ($Z\left(t_{48}\right)=2Z\left(t_{24}\right)$, with *t* expressed in hours). This behavior is described by the experimental data and correctly reproduced by Model 1.

The antigen production rate ($k_{G}$) was calculated to be 169.67 pmol/mgprot/h (from steady-state considerations at $t_{0}$), while the total antigen elimination rate ($k_{XG}^{tot}$) was equal to 13.24 /h. Figure 2(panel (b)) shows that the predicted IPP concentration after 24 h of continuous exposition to ZA was 1460 [pmol/mgprot] (IPP(t=24)=115·IPP(t=0)), and its concentration after 48 h was considerably increased (130 times compared to the initial value). Considering the immunomodulatory effects of N−bisphosphonates, these results provide evidence that our model makes it possible to predict the effects of the drug treatment by evaluating the antigen accumulation with a prospective application in clinical practice, also predicting the efficiency of the drug treatment evaluating the release of antigen in the extracellular compartment [25,26].

### 3.2. Model 1, Pulse Experiment

**Figure 3:**
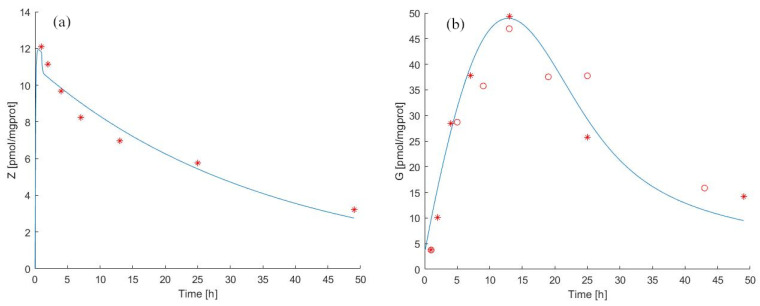
Model 1 simulation trend in the pulse experiment. Panel (**a**) shows the simulated zoledronate (ZA) trend over time; panel (**b**) shows the simulated antigen (IPP) trend over time. The continuous blue line represents the model forecast, while red asterisks represent the experimental data obtained by [39] and red circles represent the experimental data obtained by [40].

For the pulse experiment, the Model 1 forecasts for ZA and antigen concentration in cells as well as theexperimental observations from [39,40] are reported in Figure 3a,b. The estimated parameter values are shown in Table 2 and Table 4.

Pulse exposure to ZA for 1 h with 25 μM was sufficient to induce IPP accumulation after drug removal both in MCF-7 breast cancer cells (observation period 0 to 48 h) and in T47D cells (0 to 42 h). The IPP accumulation in ZA-treated T47D cells was time-dependent, reaching a maximum concentration at 12 h, as shown in [39] (9.2 pmol/mgprot) and in [40] (1052 pmol/mgprot) and gradually decreasing until end of the experiment.

We independently approximated the relationship between the rate of decay of antigen concentration in the medium and the prevalent concentration of ZA by considering Figure 2b from the published work of Benzaid et al. From the data reported in this work, it can be seen that absent ZA in the medium, the level of antigen between 24 h and 44 h decreased from around 800 to around 400 pmol/mgprot. We, therefore, could assume a spontaneous decay half-life of the antigen of approximately 20 h, corresponding to an elimination rate of approximately 3.5% absent ZA. In fact, the estimated value of the elimination rate ($k_{XG}^{tot}=2.3911$, see Table 4) is close to what can be empirically deduced from the published data, which confirms the robustness of the approach.

The transfer rate of zoledronate from the cell to the medium ($k_{YZ}$) appeared to be equal to 18.0644 /h in the pulse experiment. This value is much higher than the value estimated from the continuous experiment data. This difference might be attributed to the fact that after internalization through endocytosis, N-BPs enter the cytosol thanks to SLC37A3 and ATRAID proteins [20]. They form a lysosome complex and are responsible for releasing N-BP molecules from the lumen into the cytosol [20,44]. We can speculate that this mechanism could not be activated entirely during the short time of ZA treatment, thereby favouring the outflow of ZA from cells. This aspect could explain why the $k_{YZ}$ rate in pulse vs. continuous experiments is increased. The transport mechanisms of N-BPs are a current research topic [44], and our modelling results also suggest the need for further careful assays.

Regarding the dynamics of the IPP it is possible to note (Table 4) that the antigen production rate ($k_{G}=k_{XG}^{tot}G_{0}$) in the pulse experiment assumes a significantly lower value than in the continuous experiment.

Notice that the indicator variable $\chi_{pulse}=1$ (Equation (1)) in this experimental situation, and $t^{*}=1\;\;h$ as mentioned in MM section.

### 3.3. Model 2, Continuous Experiment

The reason for developing a second model is that the present study attempts to use in vitro observations to improve the clinical immunotherapy options for patients with advanced neoplasia. Therefore, we attempt a more faithful description of the biological phenomena while still limiting the number of equations and parameters for identifiability purposes. In particular, Model 2 expands Model 1 by considering the formation of the ZA + Enzyme + Ag complex.

**Figure 4:**
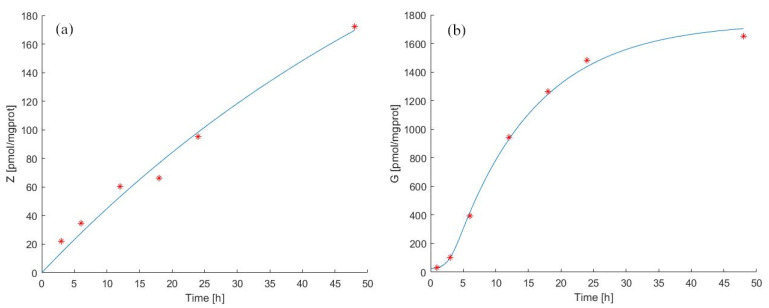
Model 2 simulation trend in the continuous experiment. Panel (**a**) shows the simulated zoledronate (ZA) trend over time; panel (**b**) shows the simulated antigen (IPP) trend over time. The continuous blue line represents the model forecast, while red asterisks represent the experimental data obtained by [39].

We thus added three parameters ($k_{BUZ}$ [/h/(pmol/mgprot)], $k_{UBG}$ [/h] and $\lambda_{UBG}$ [/(pmol/mgprot)]) and replaced the IPP concentration Equation (3) with Equations (4) and (5) to explain the kinetics of ZA and IPP considering the binding of IPP to the Enzyme+ZA complex. In this way, the equations and parameters describing ZA kinetics are identical in the two models, whereas the description of IPP dynamics differs. Model 2 forecasts in the continuous experiment for zoledronate and antigen concentration in cells, as well as the original experimental data by [39] are reported in Figure 4(panels (a) and (b)). The estimated parameter values are shown in Table 2 and Table 3.

Despite the closer approximation of Model 2 to biological reality, the general behavior of the two models is very similar, with a minor improvement in the ability of Model 2 to fit the data. We estimated $k_{G}$ (pmol/mgprot/h) to be 141.67 pmol/mgprot/h, with a total IPP decay rate ($k_{XG}^{tot}$) equal to 5.1810, lower than that estimated for Model 1. A possible explanation of this lower decay rate might lie in the retention of IPP by the enzyme/ZA complex, which is taken into account in Model 2. Indeed, part of IPP is bound to the complex and does not diffuse out of the cells into the medium. Therefore, as we have calculated, the decay rate value of IPP could be explained considering the saturation of the sites where IPP attaches to complexes within the cells.

### 3.4. Model 2, Pulse Experiment

Additionally, for Model 2, in the pulse experiment case, the observations used for the fitting procedure were those reported in [39,40]. IPP levels were observed to decrease significantly already after 24 h of treatment. This result was surprising since IPP bounds to the FPPS−ZA complex in a closed conformation, stabilizing it and leading to further sustained inhibition of FPPS [45,46]. Raikkonen and colleagues explained this data by hypothesizing that FPPS might be partly restored even in the presence of intracellular zoledronic acid, thus determining a decay of IPP.

**Figure 5:**
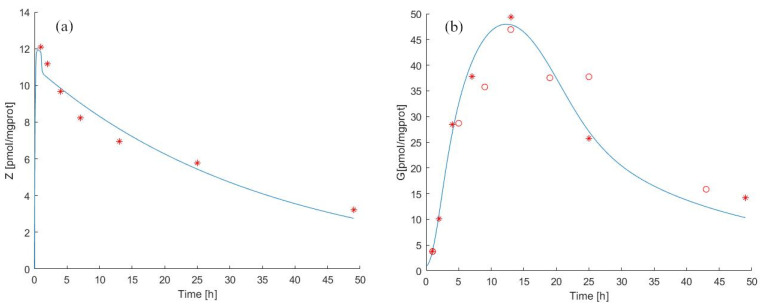
Model 2 simulation trend in the pulse experiment. Panel (**a**) shows the simulated zoledronate (ZA) trend over time; panel (**b**) shows the simulated antigen (IPP) trend over time. The continuous blue line represents the model forecast, while red asterisks represent the experimental data obtained by [39] and red circles represent the experimental data obtained by [40].

Model 2, when fitted onto data from pulse experiments, returns a $k_{XG}^{tot}$ value around 22.44 /h, which is 4.3 times larger than the corresponding value obtained from fitting continuous experiment data. We might explain this result as a consequence of the short ZA treatment and the possible leak into the cytosol. Indeed, $k_{G}$ was six times smaller in pulse than in continuous experiments. As we have described in Section 3.2 above, the short treatment interval would not favour the formation of the ATRAID-SLC37A3 complex on the lysosomes. We speculate that by missing this mechanism, the leaking of IPP might be enhanced, thus explaining the $k_{XG}tot$ and $k_{G}$ rates calculated when taking into account the FPPS/ZA/IPP complex. In fact, in Model 1, the rate $k_{XG}^{tot}$ is 5.58 times larger in the continuous than in the pulse experiment (see Figure 5).

This might indicate that our models represent the experimental conditions correctly. In any case, we consider the differences between the data obtained from the two experiments with some degree of uneasiness and would welcome a controlled repetition and re-evaluation of the pulse experiments.

### 3.5. Model Identifiability Analysis

For Model 1 and Model 2, a posteriori identifiability has been evaluated from the estimated asymptotic variance-covariance matrix of the model parameter vectors $\theta_{1}$ and $\theta_{2}$. An approximation to the variance-covariance matrix of the model parameter vector has been computed as $V=s^{2}{\left(J^{T}J\right)}^{-1}$, where $s^{2}=S\left(\hat{\theta }\right)/\left(N-q\right)$. $S(\hat{\theta })$ is the sum-of-squares loss function evaluated at the optimal θ vector; *N* is the number of observations points and *q* is the number of free parameters. The errors are assumed i.i.d. $∼N(0,\sigma^{2})$, with $s^{2}≃\sigma^{2}$.

The optimal values for the parameters are reported in Table 2 and Table 3, and the results of the identifiability analysis are reported in Table 5.

For the zoledronate kinetic model parameters ($k_{ZY}$ and $\rho_{ZY}$), the standard deviation (SD), the variation coefficients (CV) and the lower (LLC) and upper (ULC) confidence limits for the parameter suggest that the sub-model is identifiable. The parameter $k_{XY}$, being highly correlated with the couple $\left(k_{ZY},\rho_{ZY}\right)$, was excluded from the analysis, and the parameters $Y^{*+}$ and $k_{XZ}$, estimated at extremely small values, were fixed at zero.

For the antigen dynamic Model 1, the parameters $K_{G}$ and $k_{XG}^{tot}$ are determined (functions of other parameters) and were not considered in the analysis. Similarly, the parameters $k_{XGZ}$ and $G_{0}$, highly correlated with the remaining free ones, were excluded. The identifiability results for the considered parameters of Models 1 and 2 are also shown in Table 5.

The analysis results suggest that the data sets available from the literature are not sufficiently informative for reliable identification of the model parameters, in particular regarding the antigen sub-models.

## 4. Discussion and Conclusions

In this work, we developed two mathematical models (Model 1 and Model 2) to study how ZA can induce IPP accumulation in tumor cells and predict how to modulate the immune system to kill tumor cells. Increased levels of PAgs accumulate in metabolically stressed, transformed, and infected cells, which are thus sensed by Vγ9Vδ2 T cells. ZA can enhance Vγ9Vδ2 T cell’s anti-tumor functions thanks to the increasing IPP in target cells. In this regard, butyrophilin 3A1 (BTN3A1), expressed by both immune cells and tumor cells, is considered TCR-mediated sensing of phosphoAntigens (IPP). Indeed, IPP binding the intracellular domain of the protein determines a conformational change useful to be recognized by Vγ9Vδ2 TCR. Thus, BTN3A1, together with BTN2A1, are critical determinants in the recognition of human tumors by Vγ9Vδ2 T cells and trigger a cytotoxic activity. Finally, IPP is measured in the medium of cancer cell lines as a released product of treated target cells [47], and it has been proposed to bind the extracellular immunoglobulin-like domain of BTN3A1 [48] through mechanisms that are not completely clear. The generation of anti-BTN3A 20.1 mAb strongly boosts Vγ9Vδ2 T cells’ cytolytic function [49], and the newly acquired information about activation mechanisms of butyrophilin opens new perspectives in T cell-based immunotherapies. To this end, we show two models formulated by time-course measurements of ZA uptake and IPP accumulation into tumor cells from the observations of two papers, published independently and in different years [39,40]. From a mathematical point of view, both models are simple. Although Model 1 can follow the trend of the experimental data, it neglects the biologically important interaction ZA + Enzyme + IPP, which is instead considered in Model 2 without a major increase in complexity. The inhibition of FPPS by ZA results in the accumulation of the early metabolites of the mevalonate pathway, IPP [21] and its isomer DMAPP [22]. IPP becomes conjugated to adenosine-5-monophosphate (AMP) to form a novel ATP analog ApppI, while DMAPP appears to lead to the formation of ApppD. The detection of these molecules is not contemplated in the present models. There are no observations available on their concentration in time-course assays, but in the future, it would be relatively straightforward to expand these models, filling this gap.

Focusing the attention on ZA and IPP, the proposed models reproduce very well observations already established in several studies to describe the uptake of ZA and the following accumulation of IPP, as summarized below:

(1) ZA is taken up by macrophages and osteoclast cells in endocytic vacuoles; acidification of vacuoles seems to be required to release it into the cytoplasm.
(2) ZA inhibits FPP synthase in the mevalonate pathway through conformational changes in the enzyme upon binding of ZA.
(3) A second conformational change after the binding of the second substrate, IPP, forms a tightly bound inhibition complex that provides further stabilisation of the FPPS and ZA complex [45].

**Figure 6:**
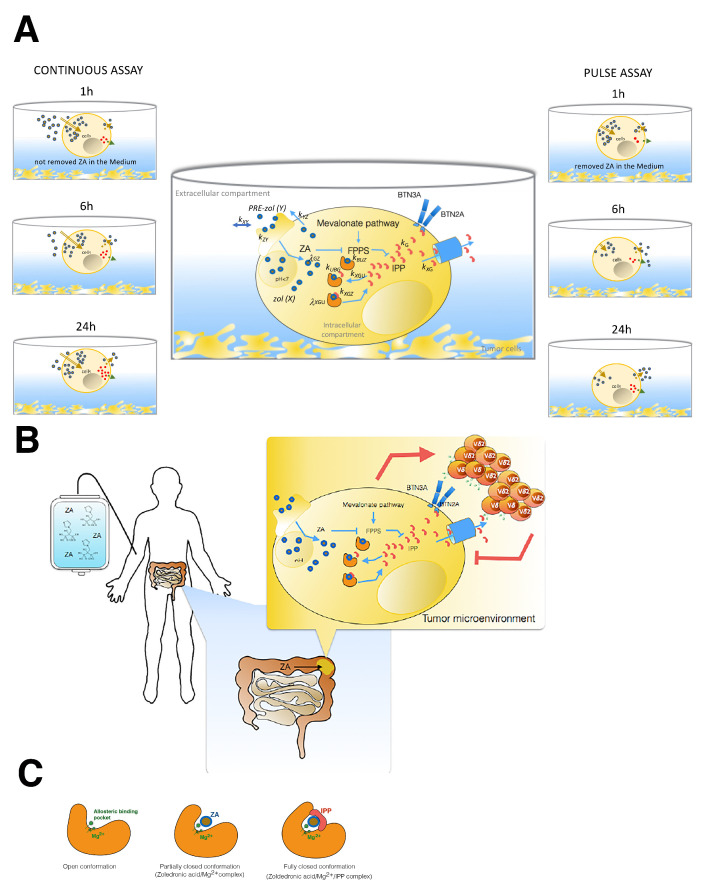
(**A**) Snapshot of the two different assays used by Raikkonen and Benzaid, from which we have extracted data to formulate our mathematical models. On the left is shown the continuous assay in which tumor cells underwent treatment for the whole time of the experiment. On the right is shown the pulse experiment performed by both authors with two different tumor target cells (MCF7, T27D); in this case, the drug was removed after 1 h via medium replacement. In the central part of the figure, we represented zoledronic acid (ZA) pharmacodynamics and IPP into the tumor cells. All process steps are characterized by the calculated parameters in Models 1 and 2. ZA inhibits FPP synthase in the mevalonate pathway through a conformational change in the enzyme. A second conformational change, after the binding of the second substrate, forms a tightly bound inhibition complex that provides a further stabilisation of the FPPS and ZA complex, resulting in the IPP accumulation. The extracellular and intracellular compartments were defined through the cellular borderline. The ZA concentration is proportional to the observations by GS-MS in the intracellular compartment. In each well, the surnatant volume was the extracellular compartment. (**B**) Impact of ZA concentration and exposure time of tumor cells on Vγ9Vδ2 T cell immune response. In this cartoon, a patient is represented during intravenous ZA infusion, and the possible effects on his tumor cells localised in the gut are also represented. We aim to translate the knowledge acquired by our models on in vitro cell experiments onto patients by focusing on the events occurring in tumor cells upon long treatment of ZA. Finally, we show that IPP accumulation acts on the activation and proliferation of γδ T cells, promoting their anti-tumoral function. (**C**) Illustration of the inhibition mechanism of ZA on FPP synthase determined by a conformational change of enzyme. The first conformation shows in the a pocket site the Mg${}^{2+}$ involved in the ZA binding. After this opened conformation follows a partially closed conformation, in which the catalytic site is occupied by ZA. The last conformation is the most stable thank to the IPP binding that determines the closed and inactive function of the enzyme [45,50].

The associated conformational change is measured as an isomerization constant (Kisom) [46] and explains ZA’s exceptional potency. Indeed, to inhibit FPPS, only small amounts of internalised ZA in the cytosol are needed [16]. The variation over time in the inhibition of the enzyme by ZA is not explicitly taken into account in the first model since this does not represent enzyme kinetics in detail. Conversely, the second model considers the bound/unbound conformation of the enzyme and can account for the variable time-course of its inhibition by ZA. A more precise model, focusing on the three conformational structures of FPPS (Figure 6C), could be designed, and, in theory, it would be possible to identify its parameters if specific data were available, detailing the crystal structure analysis and the MS analysis of FPPS complexed with ZA.

Our simple mathematical models fill a gap in the literature about the quantitative study of the effects of ZA in the generation of IPP into tumor cells. A quantitative study would be essential to predict the most effective treatment modality in cell-based immunotherapy.

While the zoledronate kinetic sub-model equations are the same for both Model 1 and Model 2, different parameter values are still obtained for the continuous and pulse experiments. In particular, it is interesting to note that cellular zoledronate is only transferred into the medium (Y(t)) and is not diminished by any other mechanism. The ZA degradation rate within the cell ($k_{XZ}$) is, in fact, estimated to be essentially zero for both models and experiments. The pre-zoledronate acid degradation rate is non-zero but assumes very small values in both Model 1 and Model 2. Another expected result is that the residue of zoledronate in the supernatant after rinsing ($Y^{*+}$), which is not considered in the continuous experiment, is zero in the pulse experiment. This result involves a jump in the time course of Y(t) equal to the value that it assumes at $t^{*}$.

The parameter $k_{YZ}$, which represents the transfer rate from compartment Z to Y, is significantly different in two experimental situations, and in the pulse experiment, is estimated at an unlikely value.

For both models, the ZA kinetics in the continuous experiment follows the data very well, while the forecast trend of the Z(t) concentration in the pulse experiment fails to capture the initial peak, which is visible in the literature data (Figure 3 and Figure 5a).

It is interesting to note that Raikkonen and colleagues repeated the pulse experiment in 2011 [51], changing the modality of treatment (starvation of serum for 18 h before ZA treatment) and the incubation time (3 h instead of 1 h). This may suggest the possibility that the authors were not completely satisfied with the original experimental procedure and why some aspects of it, not explicit and not incorporated in the model, may underlie the different dynamics of ZA uptake in the two assays. Finally, the analytic methods used have a lower detection limit for ZA and IPP concentrations, which may determine some unappreciated errors that could not be added to the model.

Regarding the Ag dynamics sub-model, while the formulation differs between Model 1 and Model 2, both models can still follow the data well, both for continuous and pulse experiments.

Model 2 considers the ZA + enzyme + Ag complex formation and can be considered more representative of the real biological processes. To maintain a biological correspondence between two G(t) models equations, the same parameter for the total IPP elimination rate ($k_{XG}^{tot}$) was used, although its computation is model-specific. In particular, $k_{XG}^{tot}$ was evaluated as the sum of two different terms: the first term, the irreducible antigen elimination rate ($k_{XG}$), is equal in both formulations; the second term in Model 1 represents the antigen elimination rate that can be suppressed in the presence of ZA ($k_{XGZ}$), and in Model 2, it represents the antigen elimination rate per percentage of an unbound enzyme ($k_{XGU}$). The numerical evaluations for $k_{XG}^{tot}$ in two models for pulse and continuous experiments are reported in Table 3 and Table 4.

While models of the response to therapy and the dynamical models for body distribution have already been reported for ZA [36,52], a model of the dynamics of cellular response to ZA has not yet been formulated to the best of our knowledge. In clinical cancer, the effect of ZA on metastatic bone disease is mostly related to its ability to decrease osteoclast-mediated bone resorption and its direct anti-tumor activity. Thus, a mathematical model that incorporates the dynamics of cellular response to ZA and the accumulation of IPP appears to be needed.

Our models provide a tool to predict the effects of ZA treatment on the accumulation of IPP, focusing attention on the timing of the accumulation to better perform γδ T cell expansion assays applied to immunotherapeutic procedures. The importance of this issue is made evident by a recent publication [38], in which the authors optimised the protocol to expand efficient Vγ9Vδ2 T cells by restimulating short-term-expanded γδ T cells with phospho-modified Vitamin C (pVC). Until now, the aim these authors considered in their studies (in vivo on mouse models or through biological samples such as urine and plasma) was to examine the relationships between dose and safety to support the clinical dosing schedule of zoledronic acid in patients with bone metastases from a variety of primary cancers [17]. In our case, instead, we look at tumor cells and their ability to uptake the drug and accumulate IPP, in each case providing a benefit that can be transferred to the patients (see Figure 6A,B). In γδ T cell-based immunotherapy, using this model, we may eventually predict how many activated and expanded circulating γδ T cells from patients with tumors could be re-activated against neoplastic cells. ZA is a drug used in the clinical approach to some oncologic patients. Several studies based on clinical trials using ZA in metastatic patients with different types of tumors have evaluated the association between clinical outcomes and frequency of circulating Vγ9Vδ2 T cells in peripheral blood, not always with beneficial results. Recently, improvements in MS-based technical analysis and the study of the infiltrating lymphocytes (focusing on scRNAseq or organoid systems) point toward personalized medicine. Thus, improved routine analysis of the infiltrating lymphocytes and tumor cells could make it possible to observe the efficacy of therapy by non-invasive sampling; in this context, this could include the application of our or similar models of ZA kinetics. Consequent antigen dynamics could help the clinical evaluation by associating it with a quantitative assessment of how the tumor cells of specific patients will respond to ZA, with obvious benefits or the clinical outcomes. The capacity to predict the amount of IPP obtained upon ZA treatment can further be useful to schedule how much ZA can be used in in vitro assays, possibly translating these predictions to the in vivo situation. Our models could also help to complete our knowledge of the multiphase distribution of plasmatic ZA since, initially, there is a release of the drug from bone tissue followed by an elimination phase involving almost exclusively the kidney.

While the models described are simple approximations to specific experimental procedures, this new mathematical analysis approach to this problem may have a substantial relevance in the future, considering the rapid advances currently being made in optimising immune therapy based on γδ lymphocytes.

## Figures and Tables

**Table 1:** Biological meanings of the variables used in Model 1 and Model 2.

Variable	Unit	Meaning
*Y*	μM	Pre-zoledronate concentration (zoledronate in medium)
*Z*	pmol/mg prote	Zoledronate acid concentration (in adhered cells)
*G*	pmol/mg prote	Isopentenyl pyrophosphate antigen concentration
*B*	%	Percentage of bound enzyme
*U*	%	Percentage of unbound enzyme

**Table 2:** Zoledronate kinetic model parameters.

Parameter	Unit	Kinetic Model	
		Continuous	Pulse
$k_{ZY}$	/h	0.007	0.607
$k_{XY}$	/h	0.004	0.029
$Y^{*+}$	μM	—	0
$k_{YZ}$	/h	0.001	18.06
$\rho_{ZY}$	pmol/mgprot/μM	25.46	14.91
$k_{XZ}$	/h	0	0

**Table 3:** Model 2: IPP antigen dynamics sub-model parameters.

Parameter	Unit	Model 2	
		Continuous	Pulse
$k_{G}$	pmol/mgprot/h	141.7	22.18
$k_{XG}$	/h	0.081	0
$k_{XGU}$	/h/%	0.051	0.224
$k_{XG}^{tot}$	/h	5.181	22.44
$G_{0}$	pmol/mgprot	27.32	0.988
$\lambda_{UBG}$	/(pmol/mgprot)	1.806	0.017
$k_{BUZ}$	/h/(pmol/mgprot)	0.099	0.150
$k_{UBG}$	/h	0.168	0.057

**Table 4:** Model 1: IPP antigen dynamics sub-model parameters.

Parameter	Unit	Model 1	
		Continuous	Pulse
$k_{G}$	pmol/mgprot/h	169.7	6.466
$k_{XG}$	/h	0.101	0
$k_{XGZ}$	/h	13.25	2.391
$k_{XG}^{tot}$	/h	13.35	2.391
$G_{0}$	pmol/mgprot	12.71	2.704
$\lambda_{GZ}$	/pmol/mgprot	0.195	0.377

**Table 5:** Summary of the standard deviation (SD), percent coefficient of variation (CV), and lower and upper confidence limits for the zoledronate kinetic model and IPP antigen dynamics sub-model (Model 1 and Model 2) parameters. Because the parameter $k_{XG}$ is present in both models, the notation $k_{XG}^{Mod1}$ and $k_{XG}^{Mod2}$ was used in the Table to differentiate them.

Parameter [Unit]	SD	CV	LLC	ULC
$k_{ZY}$ [/h]	0.001	19.30	0.004	0.0092
$\rho_{ZY}$ [pmol/mgprot/μM]	4.922	19.30	15.81	35.11
$k_{XG}^{Mod1}$ [/h]	0.064	62.80	−0.023	0.226
$\lambda_{GZ}$ [/pmol/mgprot]	0.258	146.3	−0.330	0.683
$k_{XG}^{Mod2}$ [/h]	0.051	63.10	−0.019	0.181
$k_{XGU}$ [/h/%]	0.525	1029	−0.977	1.080
$k_{BUZ}$ [/h/(pmol/mgprot)]	0.051	459.7	−0.791	0.988

## Data Availability

All the data used related to works [39,40] are reported in the manuscript and are available from the corresponding author on reasonable request.
